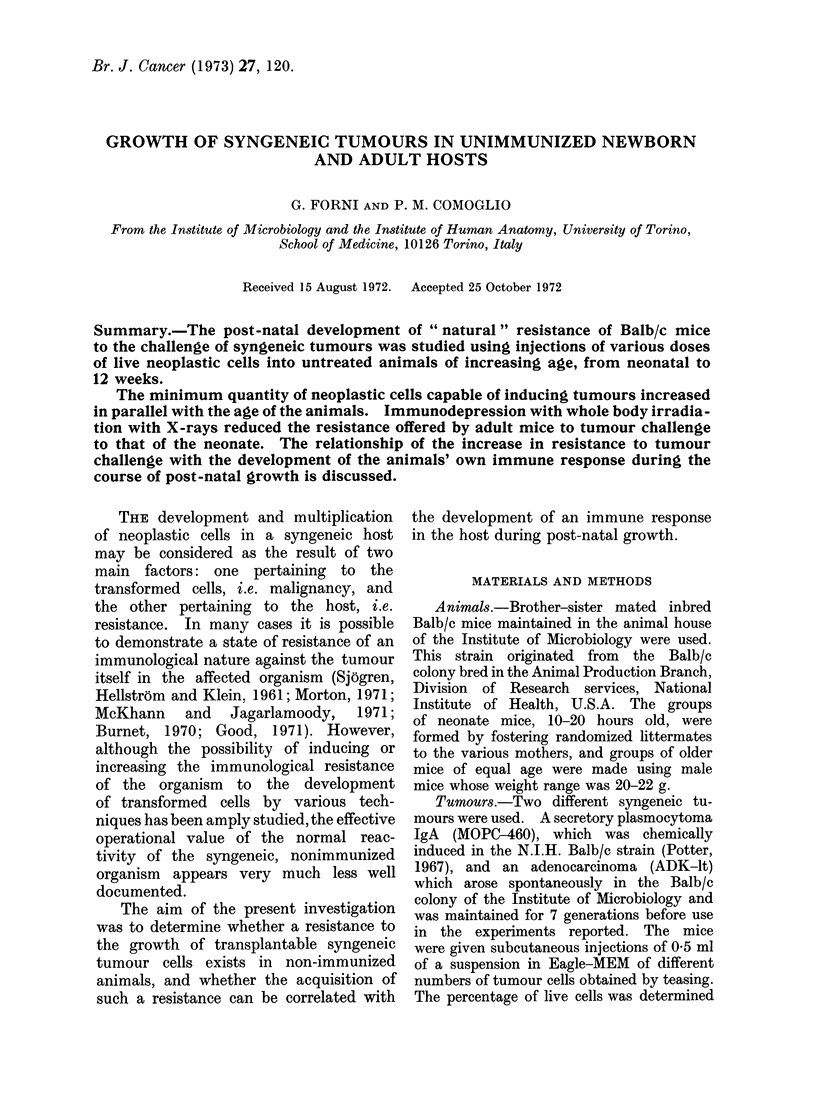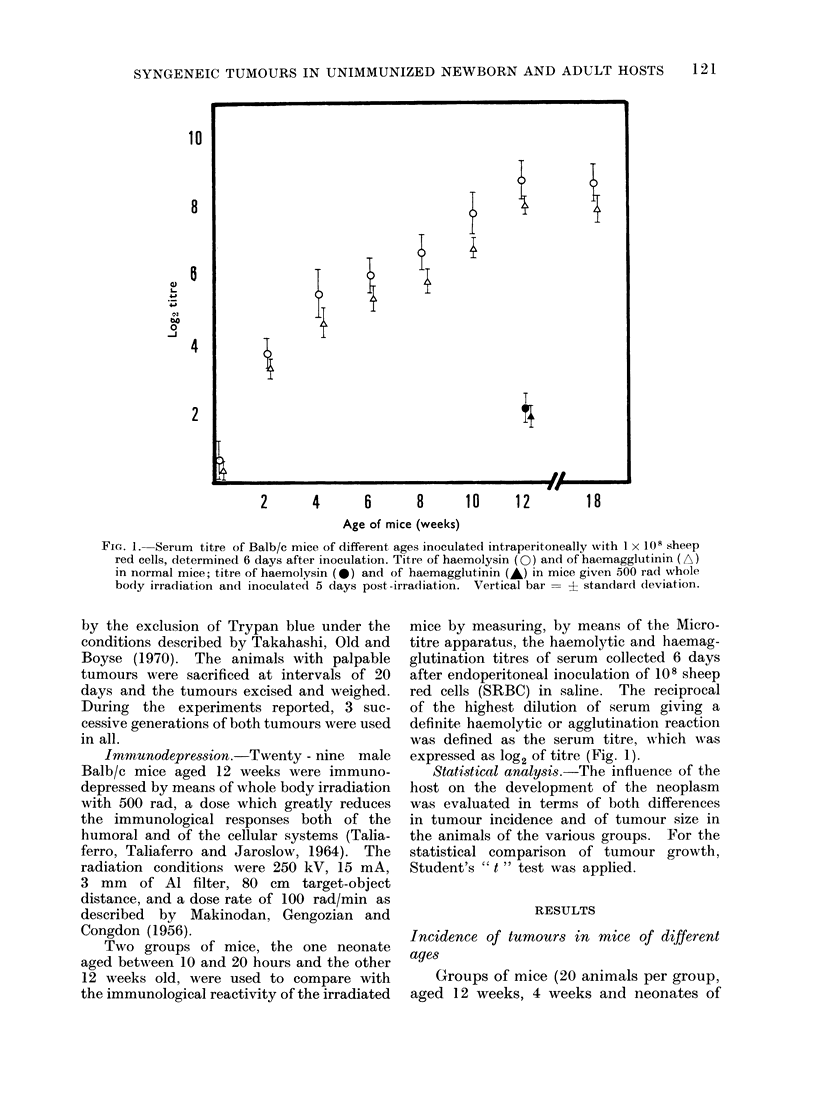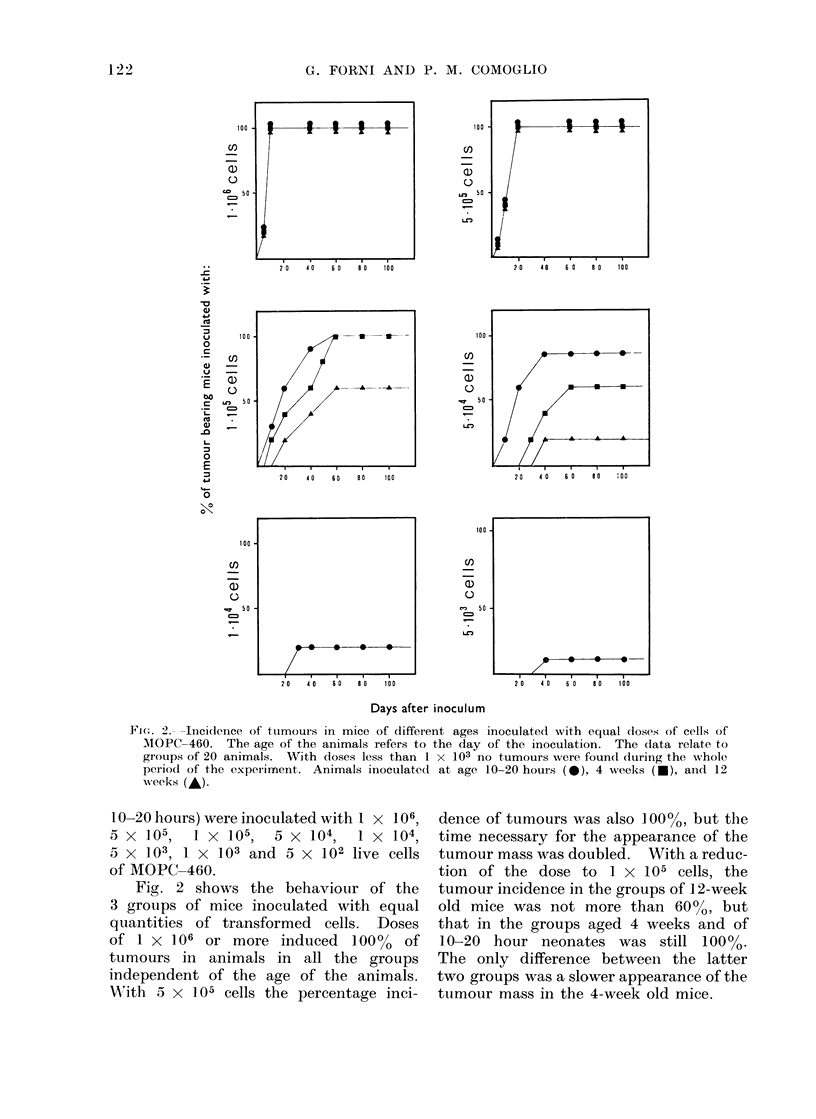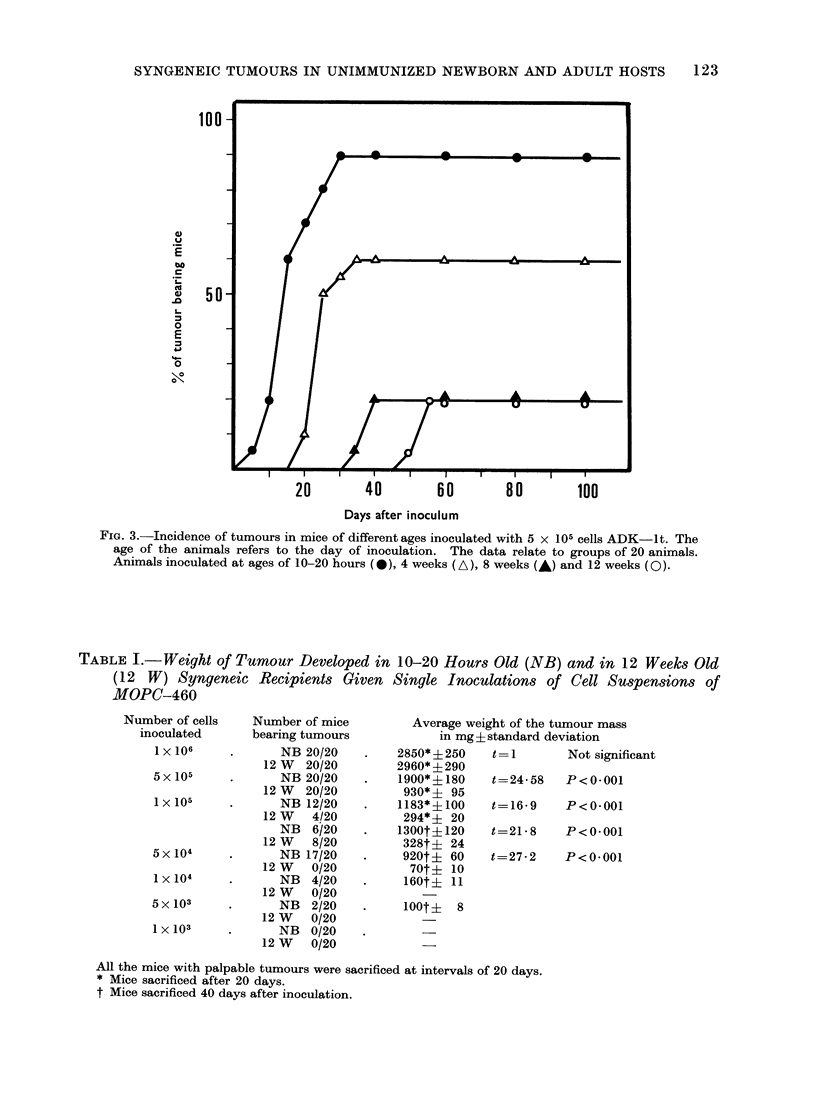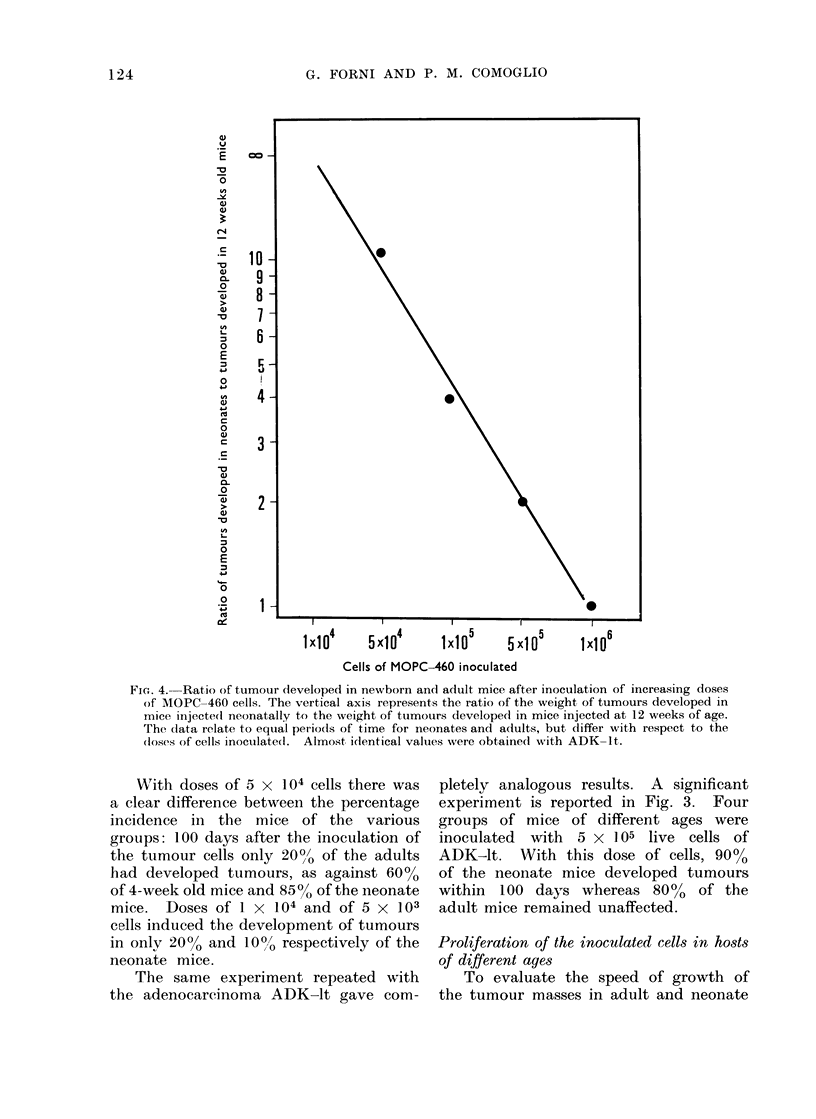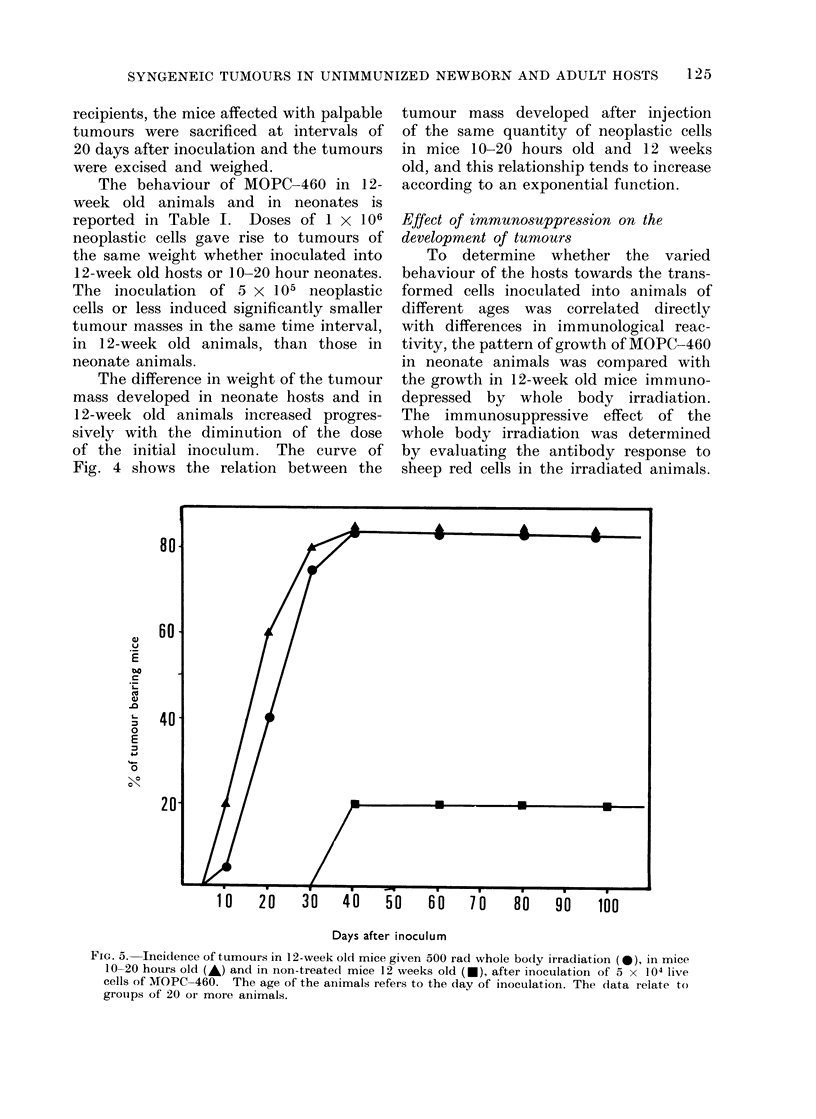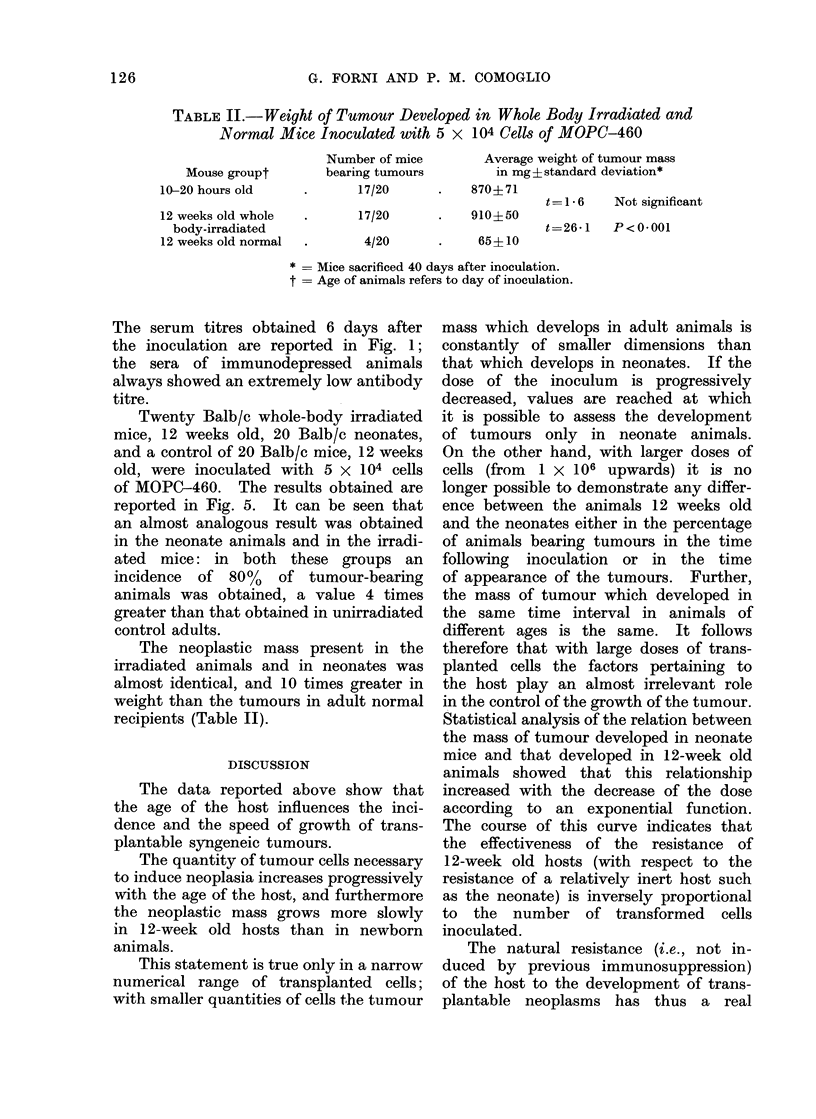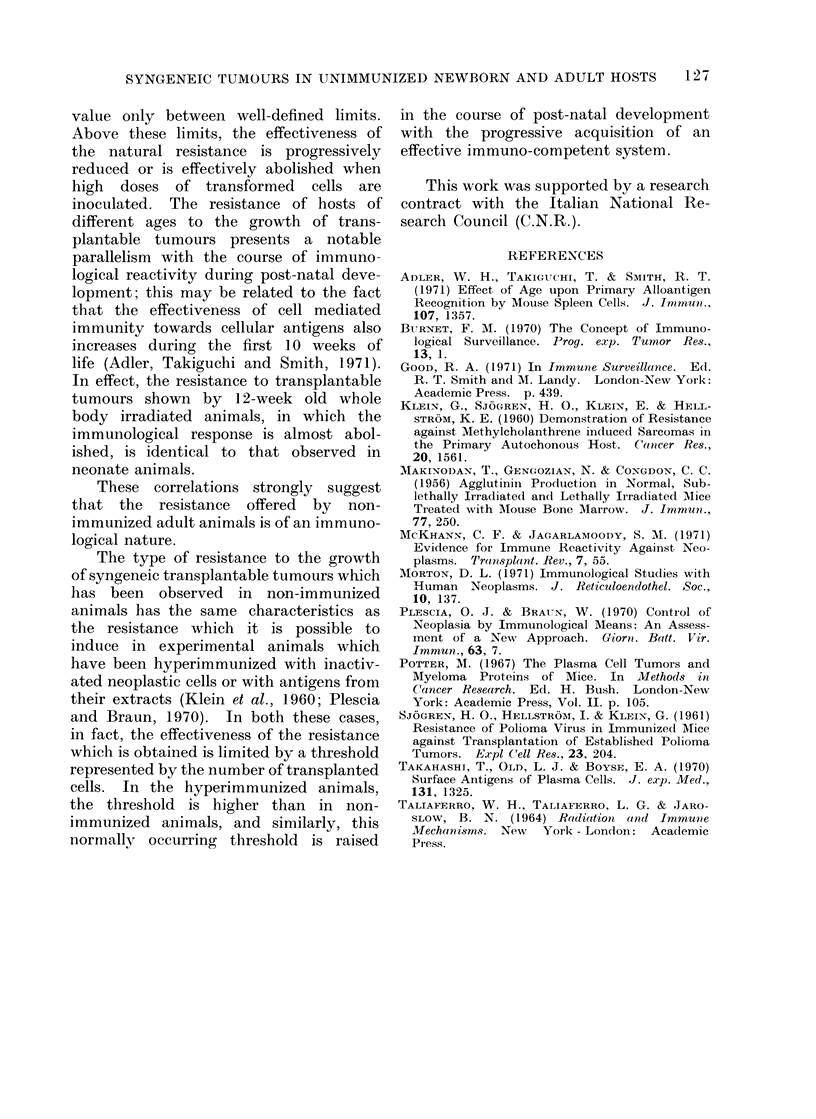# Growth of Syngeneic Tumours in Unimmunized Newborn and Adult Hosts

**DOI:** 10.1038/bjc.1973.16

**Published:** 1973-02

**Authors:** G. Forni, P. M. Comoglio

## Abstract

The post-natal development of “natural” resistance of Balb/c mice to the challenge of syngeneic tumours was studied using injections of various doses of live neoplastic cells into untreated animals of increasing age, from neonatal to 12 weeks.

The minimum quantity of neoplastic cells capable of inducing tumours increased in parallel with the age of the animals. Immunodepression with whole body irradiation with X-rays reduced the resistance offered by adult mice to tumour challenge to that of the neonate. The relationship of the increase in resistance to tumour challenge with the development of the animals' own immune response during the course of post-natal growth is discussed.


*Br. J. Cancer* (1973) **27**, 120.

### GROWTH OF SYNGENEIC TUMOURS IN UNIMMUNIZED NEWBORN AND ADULT HOSTS

G. FORNI AND P. M. COMOGLIO

*From the Institute of Microbiology and the Institute of Human Anatomy, University of Torino, School of Medicine,* 10126 *Torino, Italy*

Received 15 August 1972. Accepted 25 October 1972

**Summary.—The post-natal development of " natural " resistance of Balb/c mice to the challenge of syngeneic tumours was studied using injections of various doses of live neoplastic cells into untreated animals of increasing age, from neonatal to 12 weeks.**

**The minimum quantity of neoplastic cells capable of inducing tumours increased in parallel with the age of the animals. Immunodepression with whole body irradiation with X-rays reduced the resistance offered by adult mice to tumour challenge to that of the neonate. The relationship of the increase in resistance to tumour challenge with the development of the animals' own immune response during the course of post-natal growth is discussed.**

THE development and multiplication of neoplastic cells in a syngeneic host may be considered as the result of two main factors: one pertaining to the transformed cells, *i.e.* malignancy, and the other pertaining to the host, *i.e.* resistance. In many cases it is possible to demonstrate a state of resistance of an immunological nature against the tumour itself in the affected organism (Sjögren, Hellström and Klein, 1961; Morton, 1971; McKhann and Jagarlamoody, 1971; Burnet, 1970; Good, 1971). However, although the possibility of inducing or increasing the immunological resistance of the organism to the development of transformed cells by various techniques has been amply studied, the effective operational value of the normal reactivity of the syngeneic, nonimmunized organism appears very much less well documented.

The aim of the present investigation was to determine whether a resistance to the growth of transplantable syngeneic tumour cells exists in non-immunized animals, and whether the acquisition of such a resistance can be correlated with the development of an immune response in the host during post-natal growth.

### MATERIALS AND METHODS

*Animals.*—Brother-sister mated inbred Balb/c mice maintained in the animal house of the Institute of Microbiology were used. This strain originated from the Balb/c colony bred in the Animal Production Branch, Division of Research services, National Institute of Health, U.S.A. The groups of neonate mice, 10–20 hours old, were formed by fostering randomized littermates to the various mothers, and groups of older mice of equal age were made using male mice whose weight range was 20–22 g.

*Tumours.*—Two different syngeneic tumours were used. A secretory plasmocytoma IgA (MOPC-460), which was chemically induced in the N.I.H. Balb/c strain (Potter, 1967), and an adenocarcinoma (ADK-lt) which arose spontaneously in the Balb/c colony of the Institute of Microbiology and was maintained for 7 generations before use in the experiments reported. The mice were given subcutaneous injections of 0·5 ml of a suspension in Eagle–MEM of different numbers of tumour cells obtained by teasing. The percentage of live cells was determined

FIG. 1.—Serum titre of Balb/c mice of different ages inoculated intraperitoneally with $1 \times 10^8$ sheep red cells, determined 6 days after inoculation. Titre of haemolysin (○) and of haemagglutinin (△) in normal mice; titre of haemolysin (●) and of haemagglutinin (▲) in mice given 500 rad whole body irradiation and inoculated 5 days post-irradiation. Vertical bar = ± standard deviation.

by the exclusion of Trypan blue under the conditions described by Takahashi, Old and Boyse (1970). The animals with palpable tumours were sacrificed at intervals of 20 days and the tumours excised and weighed. During the experiments reported, 3 successive generations of both tumours were used in all.

*Immunodepression*.—Twenty - nine male Balb/c mice aged 12 weeks were immunodepressed by means of whole body irradiation with 500 rad, a dose which greatly reduces the immunological responses both of the humoral and of the cellular systems (Taliaferro, Taliaferro and Jaroslow, 1964). The radiation conditions were 250 kV, 15 mA, 3 mm of Al filter, 80 cm target-object distance, and a dose rate of 100 rad/min as described by Makinodan, Gengozian and Congdon (1956).

Two groups of mice, the one neonate aged between 10 and 20 hours and the other 12 weeks old, were used to compare with the immunological reactivity of the irradiated mice by measuring, by means of the Microtitre apparatus, the haemolytic and haemagglutination titres of serum collected 6 days after endoperitoneal inoculation of $10^8$ sheep red cells (SRBC) in saline. The reciprocal of the highest dilution of serum giving a definite haemolytic or agglutination reaction was defined as the serum titre, which was expressed as $\log_2$ of titre (Fig. 1).

*Statistical analysis*.—The influence of the host on the development of the neoplasm was evaluated in terms of both differences in tumour incidence and of tumour size in the animals of the various groups. For the statistical comparison of tumour growth, Student's "*t*" test was applied.

### RESULTS

### *Incidence of tumours in mice of different ages*

Groups of mice (20 animals per group, aged 12 weeks, 4 weeks and neonates of

FIG. 2.—Incidence of tumours in mice of different ages inoculated with equal doses of cells of MOPC–460. The age of the animals refers to the day of the inoculation. The data relate to groups of 20 animals. With doses less than $1 \times 10^3$ no tumours were found during the whole period of the experiment. Animals inoculated at age 10–20 hours (●), 4 weeks (■), and 12 weeks (▲).

10–20 hours) were inoculated with $1 \times 10^6$, $5 \times 10^5$, $1 \times 10^5$, $5 \times 10^4$, $1 \times 10^4$, $5 \times 10^3$, $1 \times 10^3$ and $5 \times 10^2$ live cells of MOPC–460.

Fig. 2 shows the behaviour of the 3 groups of mice inoculated with equal quantities of transformed cells. Doses of $1 \times 10^6$ or more induced 100% of tumours in animals in all the groups independent of the age of the animals. With $5 \times 10^5$ cells the percentage incidence of tumours was also 100%, but the time necessary for the appearance of the tumour mass was doubled. With a reduction of the dose to $1 \times 10^5$ cells, the tumour incidence in the groups of 12-week old mice was not more than 60%, but that in the groups aged 4 weeks and of 10–20 hour neonates was still 100%. The only difference between the latter two groups was a slower appearance of the tumour mass in the 4-week old mice.

FIG. 3.—Incidence of tumours in mice of different ages inoculated with 5 × $10^5$ cells ADK—1t. The age of the animals refers to the day of inoculation. The data relate to groups of 20 animals. Animals inoculated at ages of 10–20 hours (●), 4 weeks (△), 8 weeks (▲) and 12 weeks (○).

TABLE I.—*Weight of Tumour Developed in* 10–20 *Hours Old* (*NB*) *and in* 12 *Weeks Old* (12 *W*) *Syngeneic Recipients Given Single Inoculations of Cell Suspensions of MOPC*–460

| Number of cells inoculated | Number of mice bearing tumours | Average weight of the tumour mass in mg ± standard deviation | | |
|---|---|---|---|---|
| 1 × $10^6$ | NB 20/20 | 2850* ± 250 | $t=1$ | Not significant |
| | 12 W 20/20 | 2960* ± 290 | | |
| 5 × $10^5$ | NB 20/20 | 1900* ± 180 | $t=24 \cdot 58$ | $P<0 \cdot 001$ |
| | 12 W 20/20 | 930* ± 95 | | |
| 1 × $10^5$ | NB 12/20 | 1183* ± 100 | $t=16 \cdot 9$ | $P<0 \cdot 001$ |
| | 12 W 4/20 | 294* ± 20 | | |
| | NB 6/20 | 1300† ± 120 | $t=21 \cdot 8$ | $P<0 \cdot 001$ |
| | 12 W 8/20 | 328† ± 24 | | |
| 5 × $10^4$ | NB 17/20 | 920† ± 60 | $t=27 \cdot 2$ | $P<0 \cdot 001$ |
| | 12 W 0/20 | 70† ± 10 | | |
| 1 × $10^4$ | NB 4/20 | 160† ± 11 | | |
| | 12 W 0/20 | — | | |
| 5 × $10^3$ | NB 2/20 | 100† ± 8 | | |
| | 12 W 0/20 | — | | |
| 1 × $10^3$ | NB 0/20 | — | | |
| | 12 W 0/20 | — | | |

All the mice with palpable tumours were sacrificed at intervals of 20 days.
* Mice sacrificed after 20 days.
† Mice sacrificed 40 days after inoculation.

FIG. 4.—Ratio of tumour developed in newborn and adult mice after inoculation of increasing doses of MOPC–460 cells. The vertical axis represents the ratio of the weight of tumours developed in mice injected neonatally to the weight of tumours developed in mice injected at 12 weeks of age. The data relate to equal periods of time for neonates and adults, but differ with respect to the doses of cells inoculated. Almost identical values were obtained with ADK–1t.

With doses of $5 \times 10^4$ cells there was a clear difference between the percentage incidence in the mice of the various groups: 100 days after the inoculation of the tumour cells only 20% of the adults had developed tumours, as against 60% of 4-week old mice and 85% of the neonate mice. Doses of $1 \times 10^4$ and of $5 \times 10^3$ cells induced the development of tumours in only 20% and 10% respectively of the neonate mice.

The same experiment repeated with the adenocarcinoma ADK–lt gave completely analogous results. A significant experiment is reported in Fig. 3. Four groups of mice of different ages were inoculated with $5 \times 10^5$ live cells of ADK–lt. With this dose of cells, 90% of the neonate mice developed tumours within 100 days whereas 80% of the adult mice remained unaffected.

*Proliferation of the inoculated cells in hosts of different ages*

To evaluate the speed of growth of the tumour masses in adult and neonate recipients, the mice affected with palpable tumours were sacrificed at intervals of 20 days after inoculation and the tumours were excised and weighed.

The behaviour of MOPC–460 in 12-week old animals and in neonates is reported in Table I. Doses of $1 \times 10^6$ neoplastic cells gave rise to tumours of the same weight whether inoculated into 12-week old hosts or 10–20 hour neonates. The inoculation of $5 \times 10^5$ neoplastic cells or less induced significantly smaller tumour masses in the same time interval, in 12-week old animals, than those in neonate animals.

The difference in weight of the tumour mass developed in neonate hosts and in 12-week old animals increased progressively with the diminution of the dose of the initial inoculum. The curve of Fig. 4 shows the relation between the tumour mass developed after injection of the same quantity of neoplastic cells in mice 10–20 hours old and 12 weeks old, and this relationship tends to increase according to an exponential function.

*Effect of immunosuppression on the development of tumours*

To determine whether the varied behaviour of the hosts towards the transformed cells inoculated into animals of different ages was correlated directly with differences in immunological reactivity, the pattern of growth of MOPC–460 in neonate animals was compared with the growth in 12-week old mice immunodepressed by whole body irradiation. The immunosuppressive effect of the whole body irradiation was determined by evaluating the antibody response to sheep red cells in the irradiated animals.

Fig. 5.—Incidence of tumours in 12-week old mice given 500 rad whole body irradiation (●), in mice 10–20 hours old (▲) and in non-treated mice 12 weeks old (■), after inoculation of $5 \times 10^4$ live cells of MOPC–460. The age of the animals refers to the day of inoculation. The data relate to groups of 20 or more animals.

TABLE II.—*Weight of Tumour Developed in Whole Body Irradiated and Normal Mice Inoculated with* 5 × 10⁴ *Cells of MOPC*–460

| Mouse group† | Number of mice bearing tumours | Average weight of tumour mass in mg ± standard deviation* | | |
|---|---|---|---|---|
| 10–20 hours old | 17/20 | 870 ± 71 | | |
| | | | $t = 1 \cdot 6$ | Not significant |
| 12 weeks old whole body-irradiated | 17/20 | 910 ± 50 | | |
| | | | $t = 26 \cdot 1$ | $P < 0 \cdot 001$ |
| 12 weeks old normal | 4/20 | 65 ± 10 | | |

* = Mice sacrificed 40 days after inoculation.
† = Age of animals refers to day of inoculation.

The serum titres obtained 6 days after the inoculation are reported in Fig. 1; the sera of immunodepressed animals always showed an extremely low antibody titre.

Twenty Balb/c whole-body irradiated mice, 12 weeks old, 20 Balb/c neonates, and a control of 20 Balb/c mice, 12 weeks old, were inoculated with $5 \times 10^4$ cells of MOPC–460. The results obtained are reported in Fig. 5. It can be seen that an almost analogous result was obtained in the neonate animals and in the irradiated mice: in both these groups an incidence of 80% of tumour-bearing animals was obtained, a value 4 times greater than that obtained in unirradiated control adults.

The neoplastic mass present in the irradiated animals and in neonates was almost identical, and 10 times greater in weight than the tumours in adult normal recipients (Table II).

### DISCUSSION

The data reported above show that the age of the host influences the incidence and the speed of growth of transplantable syngeneic tumours.

The quantity of tumour cells necessary to induce neoplasia increases progressively with the age of the host, and furthermore the neoplastic mass grows more slowly in 12-week old hosts than in newborn animals.

This statement is true only in a narrow numerical range of transplanted cells; with smaller quantities of cells the tumour mass which develops in adult animals is constantly of smaller dimensions than that which develops in neonates. If the dose of the inoculum is progressively decreased, values are reached at which it is possible to assess the development of tumours only in neonate animals. On the other hand, with larger doses of cells (from $1 \times 10^6$ upwards) it is no longer possible to demonstrate any difference between the animals 12 weeks old and the neonates either in the percentage of animals bearing tumours in the time following inoculation or in the time of appearance of the tumours. Further, the mass of tumour which developed in the same time interval in animals of different ages is the same. It follows therefore that with large doses of transplanted cells the factors pertaining to the host play an almost irrelevant role in the control of the growth of the tumour. Statistical analysis of the relation between the mass of tumour developed in neonate mice and that developed in 12-week old animals showed that this relationship increased with the decrease of the dose according to an exponential function. The course of this curve indicates that the effectiveness of the resistance of 12-week old hosts (with respect to the resistance of a relatively inert host such as the neonate) is inversely proportional to the number of transformed cells inoculated.

The natural resistance (*i.e.*, not induced by previous immunosuppression) of the host to the development of transplantable neoplasms has thus a real value only between well-defined limits. Above these limits, the effectiveness of the natural resistance is progressively reduced or is effectively abolished when high doses of transformed cells are inoculated. The resistance of hosts of different ages to the growth of transplantable tumours presents a notable parallelism with the course of immunological reactivity during post-natal development; this may be related to the fact that the effectiveness of cell mediated immunity towards cellular antigens also increases during the first 10 weeks of life (Adler, Takiguchi and Smith, 1971). In effect, the resistance to transplantable tumours shown by 12-week old whole body irradiated animals, in which the immunological response is almost abolished, is identical to that observed in neonate animals.

These correlations strongly suggest that the resistance offered by non-immunized adult animals is of an immunological nature.

The type of resistance to the growth of syngeneic transplantable tumours which has been observed in non-immunized animals has the same characteristics as the resistance which it is possible to induce in experimental animals which have been hyperimmunized with inactivated neoplastic cells or with antigens from their extracts (Klein *et al.*, 1960; Plescia and Braun, 1970). In both these cases, in fact, the effectiveness of the resistance which is obtained is limited by a threshold represented by the number of transplanted cells. In the hyperimmunized animals, the threshold is higher than in non-immunized animals, and similarly, this normally occurring threshold is raised in the course of post-natal development with the progressive acquisition of an effective immuno-competent system.

This work was supported by a research contract with the Italian National Research Council (C.N.R.).

### REFERENCES

Adler, W. H., Takiguchi, T. & Smith, R. T. (1971) Effect of Age upon Primary Alloantigen Recognition by Mouse Spleen Cells. *J. Immun.*, **107**, 1357.

Burnet, F. M. (1970) The Concept of Immunological Surveillance. *Prog. exp. Tumor Res.*, **13**, 1.

Good, R. A. (1971) In *Immune Surveillance.* Ed. R. T. Smith and M. Landy. London-New York: Academic Press. p. 439.

Klein, G., Sjögren, H. O., Klein, E. & Hellström, K. E. (1960) Demonstration of Resistance against Methylcholanthrene induced Sarcomas in the Primary Autochonous Host. *Cancer Res.*, **20**, 1561.

Makinodan, T., Gengozian, N. & Congdon, C. C. (1956) Agglutinin Production in Normal, Sublethally Irradiated and Lethally Irradiated Mice Treated with Mouse Bone Marrow. *J. Immun.*, **77**, 250.

McKhann, C. F. & Jagarlamoody, S. M. (1971) Evidence for Immune Reactivity Against Neoplasms. *Transplant. Rev.*, **7**, 55.

Morton, D. L. (1971) Immunological Studies with Human Neoplasms. *J. Reticuloendothel. Soc.*, **10**, 137.

Plescia, O. J. & Braun, W. (1970) Control of Neoplasia by Immunological Means: An Assessment of a New Approach. *Giorn. Batt. Vir. Immun.*, **63**, 7.

Potter, M. (1967) The Plasma Cell Tumors and Myeloma Proteins of Mice. In *Methods in Cancer Research.* Ed. H. Bush. London-New York: Academic Press, Vol. II. p. 105.

Sjögren, H. O., Hellström, I. & Klein, G. (1961) Resistance of Polioma Virus in Immunized Mice against Transplantation of Established Polioma Tumors. *Expl Cell Res.*, **23**, 204.

Takahashi, T., Old, L. J. & Boyse, E. A. (1970) Surface Antigens of Plasma Cells. *J. exp. Med.*, **131**, 1325.

Taliaferro, W. H., Taliaferro, L. G. & Jaroslow, B. N. (1964) *Radiation and Immune Mechanisms.* New York - London: Academic Press.